# A Modified Formula for Intraocular Lens Power Calculation Based on Aphakic Refraction in a Pediatric Population

**DOI:** 10.18502/jovr.v18i1.12723

**Published:** 2023-02-21

**Authors:** Mohammad-Reza Jafarinasab, Behrooz Khosravi, Hamed Esfandiari, Sadid Hooshmandi, Kiana Hassanpour

**Affiliations:** ^1^Ophthalmic Epidemiology Research Center, Research Institute for Ophthalmology and Vision Science, Shahid Beheshti University of Medical Sciences, Tehran, Iran; ^2^Ophthalmic Research Center, Research Institute for Ophthalmology and Vision Science, Shahid Beheshti University of Medical Sciences, Tehran, Iran; ^3^Department of Ophthalmology, Northwestern University Feinberg School of Medicine, Chicago, IL, USA

**Keywords:** Aphakic Refraction, Intraocular Lens (IOL), IOL Power Calculation, Pediatric Cataract

## Abstract

**Purpose:**

To investigate and optimize the accuracy of aphakic refraction (AR) techniques for secondary intraocular lens (IOL) power calculation in aphakic children.

**Methods:**

Thirty-three aphakic eyes of 18 patients who were candidates for secondary IOL implantation were enrolled in the present study. Axial length (AL) measured by optical biometry was used in the biometric formula (SRK-T, Holladay II, and Hoffer-Q). AR and spherical equivalent (SE) were used in two AR-based formulas (Ianchulev, Leccissotti). True power was calculated based on postoperative SE at three months' follow-up.

**Results:**

Regarding the postoperative SE, 13 (40%) eyes were within ± 1.00 diopters (D) and 22 (66%) were within ± 2.00 D. Median absolute error (MedAE) was predicted to be 4.4 and 7.3 D with the use of Ianchulev and Leccissotti formulas, respectively. The corresponding value was 0.8 D with the biometric formula. All eyes were deemed to have myopic refraction when using the AR-based formulas except one eye with the Ianchulev formula. The coefficient of our modified formula was 1.7 instead of 2.01 in the Ianchulev formula. MedAE with the use of new formulae was 0.5 D and was comparable with the true IOL power (*P* = 0.22).

**Conclusion:**

Both Ianchulev and Leccissotti formulas resulted in a significant myopic surprise in aphakic children aged between 4.5 and 14 years. The modified formula proved to determine a more accurate SE that is comparable with biometric formulas.

##  INTRODUCTION

Intraocular lens (IOL) power calculation remains a challenging issue in the pediatric population.^[1]^ There are two methods of IOL power calculation that are most commonly used in aphakic children including the use of either biometric formulas or refractive vergence formulas. Anatomical measurements including axial length (AL) and keratometry (K) are used in the biometric method formulas such as Sanders–Retzlaff–Kraff (SRK-T),^[2]^ Holladay II,^[3]^ or Hoffer-Q.^[4]^ In aphakic refraction (AR)-based formulas, AR is applied to measure the power of the IOL.^[5]^ The data of AL and K are not always available. AR could be used either preoperatively or- intra- and postoperatively, for both primary and secondary IOL calculations in adults and children. When using this AR method of measurement intraoperatively for primary IOL implantation, the anterior chamber should be formed after performing the lensectomy to refract the aphakic eye using either a portable auto refractometer or retinoscopy. The spherical equivalent (SE) could then be placed in the formula without further need of the AL and K measurements.

Currently, there are several available AR-based formulas including the Hug,^[6]^ Khan,^[7]^ Ianchulev,^[8]^ and Leccissotti^[9]^ formulas. In Khan's formula, AL is calculated based on the AR, and K is assumed to be 44.^[7]^ Ianchulev et al^[8]^ have introduced a formula that does not include AL and K measurements compared favorably with the biometric IOL power calculation. Subsequently, Leccissotti used aphakic SE in a personal formula for high myopic patients as well as in the Ianchulev formula for low myopic patients and reported a parabolic relationship between the SE and IOL power.^[9]^ Wong et al^[10]^ investigated the accuracy of the Iaunchulev and Leccissotti formulas in 182 eyes of adult patients undergoing cataract surgery. The authors found that the Ianchulev formula could be applied in all eyes except in those experiencing high myopia while the Leccissotti formula worked particularly poorly in short eyes but performed better in eyes with myopia.

In recent years, intraoperative wavefront aberrometry with Optiwave Refrctive Analyzer (ORA) system has shown comparable postoperative refractive outcomes when compared to conventional biometry (IOL Master) in adult patients who underwent routine cataract surgery. However, its use in the pediatric population is yet to be determined.^[11]^

The application of refractive vergence formulas in the pediatric population remains a controversial issue. Abdel-Aziz et al^[12]^ compared Khan's and Hug's formulas with the Holladay I formula and found a 0.8 D reduction in the accuracy of the refractive vergence formula. Similarly, Nakhli et al reported better performance with the AL vergence formula compared to the refractive vergence formula.^[13]^

The current study is designed to investigate the accuracy of two refractive vergence formulas in secondary IOL calculations in children as well as the clinical outcomes when modifying the AR formulas to determine more accurate predictive results.

##  METHODS

Thirty-three aphakic eyes of 18 patients who were candidates of secondary IOL implantation aged 4.5–14 years were all enrolled in this comparative case-series between October 2013 and September 2019. The exclusion criterion was a cornea that was too hazy for refraction. The study protocol was approved by the Ethics Committee of Shahid Beheshti University of Medical Sciences. The study adhered to the tenets of the Declaration of Helsinki and written informed consent was obtained from the legal guardians of the patients.

### Biometric Formulas

All measurements before and after the operations were performed by an optometrist experienced in working with the pediatric population. AL and keratometry were measured using optical biometry (Lenstar LS 900, Haag-Streit AG, Switzerland). To calculate secondary IOL power, SRKT^[2]^ was used in eyes with AL measuring > 22 mm and Hoffer-Q^[4]^ was the formula of choice in eyes with AL measuring < 22 mm.

The patient's age was used to determine the target refraction. Target refraction was set for emmetropia in children older than six and 1 D of hyperopia in children younger than six years old.

### Refractive Vergence Formulas

An experienced pediatric ophthalmologist (BK) measured the AR with the use of an autorefractor (Nikon Instruments Inc., Melville, New York, USA) or retinoscope (Welch Allyn SureSight, Welch Allyn, Skaneateles Falls, New York). The mean of four SE autorefraction measurements was used in the refractive vergence formulas of Lanchulev and Lescilloti.

### Surgical Technique

All surgeries were performed by an experienced pediatric cataract surgeon (MRJ). Under general anesthesia, the main wound was created with a 2.8 mm keratome and intracameral diluted adrenaline (1/1000) was used for pupillary dilation. An ophthalmic viscoelastic device (OVD) was used to form the anterior chamber and release the synechiae. A three-piece foldable acrylic IOL (AcrySof MA60, Alcon Laboratories) was placed in the ciliary sulcus followed by irrigation and removal of the OVD. The wound was sutured with a 10-0 Nylon (Nylon, Ethicon Inc., Somerville, NJ) suture. Subconjunctival betamethasone and cefazolin were injected upon the conclusion of the surgery. Topical ciprofloxacin 0.3% (Ciplex, Sina Daru, Tehran, Iran) was used four times per day for one week while betamethasone 0.1% (Betasonate, Sinadaru, Tehran, Iran) eye drops were used four times per day and tapered off over a month.

Patients were followed on day one, week one, month one, and month three postoperatively. The refraction was measured at the third-month follow-up visit.

### Statistical Analysis

Frequency and percentages were used to report the descriptive data.

Postoperative refraction was used to estimate the “actual” IOL power; Regarding postoperative SE, the IOL power that would cause emmetropia was calculated for each subject. This value was considered as “true” IOL power. For each diopter of myopia, 1 D was reduced from the actual calculated IOL power. Similarly, for each diopter of hyperopia, 1.5 D were added to the actual calculated IOL power. The mean (Mean Absolute Error [MAE]) and median (MedAE) of the difference between true IOL power and calculated IOL powers were then calculated for each formula. All statistical analyses were performed using SPSS (IBM). A *P-*value < 0.05 was considered significant.

##  RESULTS

Thirty-three eyes of 18 patients were included in this study. Median age of the patients was 8.7 ± 2.9 years ranging from 5 to 13.5 years. Average AL was 23.3 ± 1.8 mm ranging from 18.5 to 26.6 mm. AL was > 24 in 13 eyes (39.3.5 %), between 22 and 24 mm in 14 eyes (45.4%), and < 22 mm in 6 eyes (18.8%) [Table 1].

### Biometric Formulas

**Figure 1:**
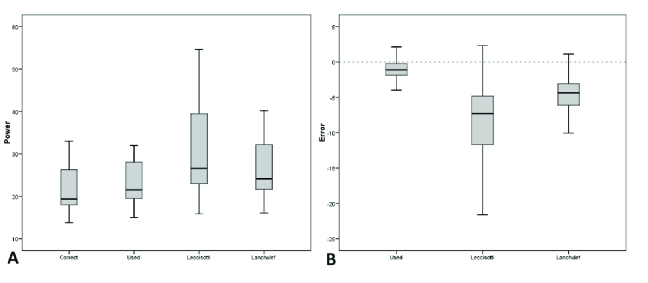
Box and whisker plot demonstrating calculated IOL powers (A) and the error using different formulas (B). Error is based on the difference between True power (Correct bar in A) and calculated powers using different formulas.

The mean preoperative SE was +13.2 D (range, +8.0 to +20) that improved to –0.9 D (range, – 3.00 to +4.00) postoperatively. Considering the multiple measurements of AL, the mean SE was –0.8, –0.98, and +0.62 D in ALs > 24, between 22 and 24, and < 22 mm, respectively. The MedAE and MAE were –0.9 ± 2 and –1.1, respectively [Table 2]. Figure 1 demonstrates the postoperative SE plotted against the preoperative SE in each patient.

### Refractive Vergence Formula

Theoretically, if the Ianchulev formula was used to assess the refractive vergence, all eyes except one would reflect myopic refraction. The mean postoperative SE would be –4.5 ± 2.6 D while one eye would have +1.50 D of hyperopic refraction. In eyes with AL > 24 mm, the mean SE would have been –3.75 D (range, –1.0 to –6.0 D). The mean SE would be –5.50 (range, –10.0 to +1.50) in AL < 24 mm.

Similarly, if the Leccissotti formula was utilized, it would have resulted in an average SE of –11.0 (range, –1.50 to –20.0) in AL < 24 and –4.85 (range, –4.85 to 8.50 D) in AL > 24 mm [Tables 2 & 3].

**Figure 2:**
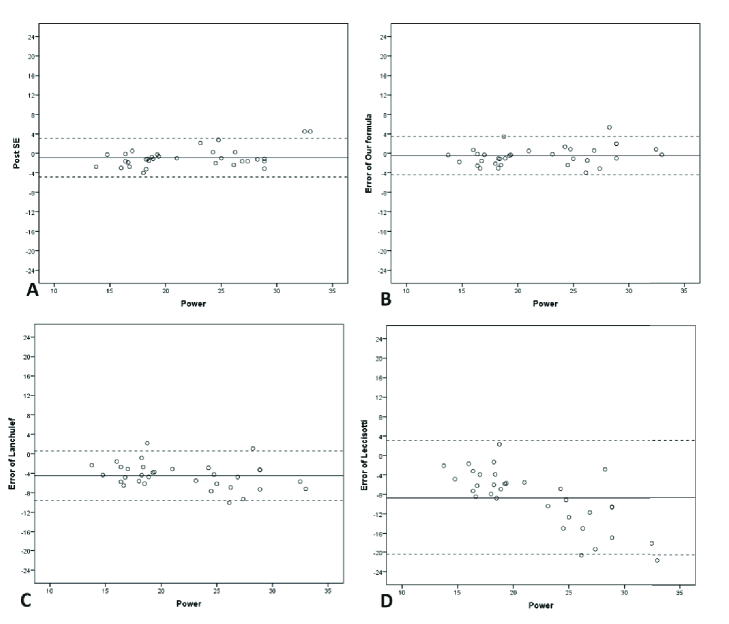
Scatter plots demonstrating postoperative spherical equivalent (SE) (A), the error of different formulas plotted against the calculated power based on the modified formula (B), Ianchulev (C), and Leccissotti (D).

With the use of the Ianchulev formula, MedAE and MAE would be 4.5 and 4.4 D, respectively. The corresponding values for the Leccissotti formula were 8.7 and 7.3 D, respectively [Figure 2].

**Table 1:** Baseline characteristics of study participants.


orangeParameter	orangeMean ± SD	orangeMedian (Range)
Age (yr)	8.7 ± 2.9	8.0 (5.0 to 13.50)
AL	23.3 ± 1.8	23.5 (18.5 to 26.6)
Preoperative sphere	13.8 ± 3.2	13.3 (8.0 to 20.0)
Preoperative cylinder	–1.0 ± 1.0	–1.0 (–3.3 to 0.8)
Preoperative SE	13.2 ± 3.2	12.3 (8.0 to 20.0)
Postoperative sphere	0.1 ± 2.0	–0.3 (–3.0 to 6.0)
Postoperative cylinder	–1.6 ± 1.0	–1.5 (–4.3 to 0.8)
Postoperative SE	–0.9 ± 2.0	–1.1 (–4.0 to 4.5)
	
	
white<bcol>3</ecol>AL, axial length; SE, spherical equivalent; yr, years

**Table 2:** Mean and median of calculated IOL power with different formulas.


orangeParameter	orangeMean	orangeMedian (Q1, Q3)	orangeMin	orangeMax
True power*	22.0 ± 5.4	19.4 (18.0, 26.3)	13.8	33.0
Biometric formulas	22.8 ± 4.9	21.5 (19.5, 28.0)	15.0	32.0
Postoperative SE	–0.9 ± 2.0	–1.1 (–1.9, –0.3)	–4.0	4.5
Leccissotti formula	30.7 ± 10.7	26.5 (23.0. 39.5)	15.9	54.6
Error	–8.7 ± 6.0	–7.3 (–11.7, –4.8)	–21.6	–1.5
Ianchulev formula	26.4 ± 6.7	24.1 (21.6, 32.3)	16.1	40.2
Error	–4.5 ± 2.6	–4.4 (–6.4, –3.1)	–10.1	1.5
Modified formula	22.5 ± 5.2	20.9 (19.3, 26.9)	14.1	33.3
Error	–0.5 ± 2.0	–0.5 (–1.8, 0.6)	–4.0	5.4
	
	
white<bcol>5</ecol>IOL, intraocular lens; Q1, first quartile; Q3, Third quartile

**Table 3:** Comparison between IOL power calculated with different formulas subtracted from the true power.


orangeStatistics	orangeUsed – True	orangeLeccissotti – True	orangeIanchulev – True	orangeModified – True
Pearson correlation	0.931	0.931	0.931	0.932
ICC	0.926	0.751	0.908	0.931
Δ Mean ± SD (Diopter)	–0.9 ± 2	–8.7 ± 6	–4.5 ± 2.6	–0.5 ± 2
95% CI	–1.58 to –0.22	–10.73 to –6.67	–5.39 to –3.61	–1.17 to 0.17
*P*-value*	< 0.001	< 0.001	< 0.001	0.116
Δ Median (range)	–1.1 (–4 to 4.5)	–7.3 (–21.6 to 2.3)	–4.4 (–10.1 to 2.2)	–0.5 (–4 to 5.4)
95% LoA	–4.82 to 3.02	–20.46 to 3.06	–9.6 to 0.6	–4.42 to 3.42
	
	
white<bcol>5</ecol>*Based on Paired *t*-test True indicates the calculated power based on postoperative refraction Correlation of eyes was considered in the calculation of SD, 95% CI, and LoA IOL, intraocular lens; ICC, intra cluster correlation; Δ , inter-formula difference; CI, confidence interval; LOA, limits of agreement

**Table 4:** Achieved refraction with the use of different formulae.


orange**Achieved SE (D)**	orange**Used formula**	orange**Ianchulev formula**	orange**Leccissotti formula**	orange**Modified formula**
Within ± 0.5	6 (18.2 %)	0 (0%)	0 (0%)	7 (21.2 %)
Within ± 1	13 (39.4 %)	2 (6.1%)	0 (0%)	19 (57.6 %)
Within ± 2	22 (66.7 %)	4 (12.1%)	2 (6.1%)	27 (81.8 %)
–2 < SE or SE > +2	33 (100%)	33 (100 %)	33 (100%)	33 (100 %)
	
	
white<bcol>5</ecol>SE, spherical equivalent; D, diopter

### Modified Formula

With the step-by-step reduction of the coefficient of the Ianchulev formula from 2.01 to 1.70, the mean SE improved to –0.5 ± 2 (Median –0.5, range from –4 to 5.4) D. Twenty-two (66%) eyes would reflect myopic results, while 11 (34%) would reflect hyperopic refraction [Table 2 & Figures 1 & 2]. MedAE and MAE were 0.5 and 0.5, respectively Table 4 demonstrates the ranges of achieved refractions with the use of different formulas.

##  DISCUSSION 

The refractive vergence formulas that use AR instead of AL and keratometry could be used in assessing IOL power calculations for aphakic children. IOL power calculation in an eye that is still growing is a challenging process.

Recent advances in technologies resulted in more reliable AL, and keratometry measurements improved the ability in predicting more accurate IOL power and subsequent better visual outcome in pediatric cataract surgery. In this study, we have investigated the use of two refractive vergence formulas in comparison to conventional formulas. In this series of 33 eyes, we demonstrated that refractive vergence formulas would result in reflecting significant myopic refraction, while the conventional formulas resulted in reflecting favorable refraction within ± 0.5 D from the target refraction. Furthermore, postoperative myopic refractive error was higher in eyes with shorter AL. In high myopic patients, the Leccissotti formula was slightly closer to target refraction than the Ianchulev formula when calculated preoperatively.

Our findings are in line with previous studies on this subject, Nakhli et al^[13]^ compared the axial vergence formulas such as Hoffer-Q, SRK-T, and Holladay with the refractive vergence formulas as presented by Ianchulev, Khan, and Mackool in 31 pediatric cataract eyes. The authors reported more accurate results to target refraction with the preoperative axial vergence formulas when compared with the true IOL calculations postoperatively. The amount of error was predicted to be –5.48 ± 3.55 diopters with the use of the Ianchulev formula, which is comparable to the predictive error calculation of 4.5 ± 2.6 D observed in our study.

Our study differs from Khan and Al Gaeed's study in which AL was estimated from the AR through the use of a complicated formula.^[7]^ They used the estimated AL as well as 44.0 as a constant keratometry value in the Holladay formula. Results of their comparison confirmed the comparable accuracy of the AR pre-calculation of AL with the pre-calculated AL using the Holladay formula in 50 eyes where both formulas resulted in values that were close to the “true” IOP power calculation postoperatively.^[7]^ In Nakhli et al's study, Khan's method resulted in more accurate prediction as compared to the Ianchulev formula (–1.66 ± 3.19 vs –5.48 ± 3.55 D).^[13]^ Due to the inability to retrieve accurate measurements for AL and the limitation of using a constant to represent keratometry, we did not use this formula in our study.

In another study comparing Hug's and Khan's refractive vergence formulas, the mean error was greater by 0.8 as compared to the standard biometric methods. The mean predicted error was 2.4 ± 2.0 with both Khan's and Hug's formulas as compared to –4.5 ± 2.6 D and –8.7 ± 6.0 in the Ianchulev and Leccissotti formulas, respectively.^[12]^ Notably, the between-study comparison of predicted errors is biased due to differences in population, measurement, and surgical techniques. Therefore, the results should be interpreted with caution.

Considering the significant myopic surprise with the Ianchulev formula, we modified the current coefficient of the formula from 2.01 to 1.70. IOL power calculation with the new coefficient proved to be comparable with the biometric formula. Our modified coefficient is close to a coefficient proposed by Mackool et al.^[14]^ Mackool et al suggested the following formula in determining the IOL power in patients with post-LASIK cataract extraction:

IOL power = 1.75 * AR (SE).

Accurate biometry could be difficult in patients with a history of refractive surgery. The small difference between the Mackool coefficient and ours could be attributed to the position of the IOL. In our study, all the IOLs were placed into the sulcus in contrast to the bag implantation in Mackool's study.

There are several limitations to our study including an assessment on a small population of patients which may affect the results and also precludes a valuable analysis of the hypothetical prediction of postoperative refractions with the use of refractive vergence formulas.

In summary, the present study confirmed the superiority of the use of conventional biometric formulas in the secondary IOL power calculation in aphakic children. However, since the biometric measurements are not always available in aphakic children, the presence of a comparable refractive vergence formula is critical. We found that the use of aphakic SE multiplied by our modified coefficient of 1.7 would result in favorable clinical outcomes in aphakic children aged between 4.5 and 14 years. To determine a more accurate prediction of error, the use of this formula in conjunction with testing on an expanded population in the real world is recommended.

### Financial Support and Sponsorship

None.

### Conflicts of Interest

None.
